# Relationship between sperm quality and total fertilization failure in intracytoplasmic sperm injection and in vitro fertilization cycles: A cross-sectional study

**DOI:** 10.18502/ijrm.v20i5.11056

**Published:** 2022-06-08

**Authors:** Hassan Safari, Fatemeh Anbari, Saeed Ghasemi-Esmailabad, Behnam Maleki, Laleh Dehghan Marvast, Ali Reza Talebi

**Affiliations:** ^1^Research and Clinical Center for Infertility, Yazd Reproductive Sciences Institute, Shahid Sadoughi University of Medical Sciences, Yazd, Iran.; ^2^Department of Reproductive Biology, Shahid Sadoughi University of Medical Sciences, Yazd, Iran.; ^3^Andrology Research Center, Yazd Reproductive Sciences Institute, Shahid Sadoughi University of Medical Sciences, Yazd, Iran.

**Keywords:** Intracytoplasmic sperm injection, In vitro fertilization, Reactive oxygen species, Chromatin, DNA fragmentation.

## Abstract

**Background:**

Total fertilization failure (TFF) is associated with essential mechanistic and cellular events.

**Objective:**

The present study is a comprehensive examination of detrimental effects with well-known assays for predicting TFF in conventional in vitro fertilization (IVF) and intracytoplasmic sperm injection (ICSI) cycles.

**Materials and Methods:**

Semen parameters of 90 men, including 60 cases who had experienced IVF/ICSI failure and a control group of 30 individuals, were evaluated. Sperm chromatin/DNA quality assessments were done by aniline blue, toluidine blue, chromomycin A3, and terminal deoxynucleotidyl transferase-mediated dUTP nick end labeling (TUNEL) assays. A lipid hydroperoxide (LPO) kit was used to measure the LPO, and JC1 staining was used to evaluate mitochondrial membrane potential (MMP).

**Results:**

There were statistically significant differences found between the IVF, ICSI and control groups by the toluidine blue (p = 0.01), TUNEL (p = 0.02), and chromomycin A3 (p < 0.001) tests, but not by the aniline blue staining. Furthermore, there was a significant difference regarding LPO concentration and high MMP in cases of IVF fertilization failure compared to the control group (p = 0.04, p = 0.02, respectively). The logistic regression model showed that sperm viability was predictive for fertilization failure in the ICSI group. Sperm chromatin and DNA quality assays were not predictors for TFF in either group.

**Conclusion:**

Cellular events such as high DNA fragmentation damage, high levels of reactive oxygen species, and low MMP levels can cause TFF in IVF and ICSI programs. Diagnostic tests, especially in cases with previous fertilization failure, showed significant differences in sperm chromatin and DNA quality between groups but could not predict the risk of TFF.

## 1. Introduction

Total fertilization failure (TFF), which is the failure of fertilization in all oocytes, is an unpredictable event in assisted reproductive technology programs. It has been reported to range from 5-10% for conventional in vitro fertilization (IVF) (1) and up to 3% for intracytoplasmic sperm injection (ICSI) procedures (2). The main factor of TFF in the ICSI cycle that is associated with the failure of oocyte activation is due to defective spermatozoa, such as swollen sperm heads, deficiency of the oocyte-activating capacity of spermatozoa, and premature chromosome condensation or decondensation failure (3, 4). In contrast, penetration failure, due to problems in sperm molecular receptors, acrosomal defects, or sperm ejection, is the main cause of fertilization failure in the IVF program (5). Also, it has been demonstrated that abnormal calcium oscillations can be attributed to oocyte quality in both groups (6).

Several studies have shown that sperm DNA fragmentation and lipid hydroperoxide (LPO) cascades are caused by high reactive oxygen species (ROS) levels. This results in pathological effects on sperm function, including premature acrosome reaction, reduced mitochondrial membrane potential (MMP), LPO of the sperm membrane, and DNA fragmentation. It may also be involved in the etiology of TFF (7-9). In this regard, sperm DNA fragmentation had higher specificity (93.3%) compared to progressive motility (77.8%) in predicting the fertilization rate (10). Therefore, conventional sperm parameters are not always an appropriate predictor of fertilization results. This topic is a particularly valuable area of research for cases involving patients with unexplained infertility because the sperm parameters for these cases are normal. There are several appropriate assays for the assessment of sperm chromatin integrity and DNA damage; for example, chromomycin A3 (CMA3) staining, the terminal deoxynucleotidyl transferase-mediated dUTP nick end labeling (TUNEL) assay, together with cytochemical assays such as aniline blue (AB) staining and toluidine blue (TB) staining (11).

A previous study reported a negative association between sperm chromatin condensation, analyzed by AB and TB staining, with sperm count, normal morphology, and progressive motility. These assays were useful for assessing male fertility potential in the study and were described as simple, fast, and accessible assays (12). CMA3 staining indicates abnormal protamination so that histones will remain in the sperm nucleus during spermiogenesis. Poor chromatin packaging may correlate to sperm decondensation deficiency in IVF or ICSI cycles and can eventually lead to fertilization failure (13). However, MMP is a key indicator of mitochondrial activity, which is essential for oocyte activation and therefore fertilization success rates. Based on our knowledge, no study has included a comprehensive examination of sperm parameters, DNA, and chromatin evaluation showing different types of damage, ROS, and MMP in both ICSI and IVF failure fertilization cycles. Also, few studies have paid attention to the effect of each parameter on predicting fertilization.

The main objective of this study was to evaluate the potential value of the assays mentioned above, when seeking to predict the fertilization power of sperm. In this study, a comparison of IVF and ICSI fertilization failure was made simultaneously.

## 2. Materials and Methods

### Subjects

This cross-sectional study enrolled 90 participants with unexplained infertility who had experienced TFF referred to Yazd Reproductive Sciences Institute, Yazd, Iran between September 2019 and December 2020. Within this group, 60 individuals had experienced IVF/ICSI failure (n = 30/each) in the first cycle of treatment (these were categorized as the cases), and 30 individuals had successful fertilization after assisted reproduction procedures (these were considered as the control group). For more matching between groups, female partners with the favorable conditions of age < 38 yr, baseline follicle stimulating hormone (b-FSH) < 12 mIU/ml, anti-mullerian hormone > 1 ng/ml, body mass index between 20-25 kg/m^2^, and more than 3 cumulus-oocyte complexes were included (14). The age range of the male partners was 20-45 yr. Individuals were excluded if they had underlying diseases such as varicocele, cancer, urinary tract infection, diabetes or orchitis, or had cumulus-oocyte complexes or denudated oocytes with abnormal morphology (such as the presence of a large polar body, large perivitelline space, refractile bodies or vacuoles which increase the likelihood of TFF (15). The ICSI procedure was performed by a skilled embryologist with more than 10 yr of experience.

### Sperm collection and semen analysis

Semen samples were collected from men who had a medical history of TFF after 2-7 days of abstinence. The samples were allowed to liquefy 15-30 min at 37 C. After liquefaction, semen parameters including concentration, motility, viability, and morphology were assessed according to World Health Organization guidelines (16). The Diff-Quik rapid staining kit (Sigma-Aldrich, Germany) was used for the comprehensive assessment of the morphology.

### Stimulation protocol

Participants were stimulated with gonadotrophin-releasing hormone antagonist (CetrotideⓇ; Merck Serono, Darmstadt, Germany) and recombinant FSH (Gonal-FⓇ; Merck Serono, Switzerland) using standard protocols. After the growth of some follicles to 18 mm diameter, recombinant human chorionic gonadotrophin (OvitrelleⓇ; Merck Serono, Germany) was administered to achieve final maturation. Oocyte retrieval was done by transvaginal ultrasonography guidance after 36 hr.

### Sperm chromatin/DNA quality assessments

Standard cytochemical assays, including aniline blue (AB), toluidine blue (TB), CMA3 and TUNEL, were used to assess chromatin status and DNA integrity.

#### AB staining

Acidic dyes such as AB stains react with histone proteins containing the amino acid lysine. AB stains also detect sperm chromatin condensation anomalies related to residual histones. The smears of washed semen samples were allowed to dry in the air. Then, the slides were fixed in 3% buffered glutaraldehyde in 0.2 M phosphate buffer (pH = 7.2) for 30 min at room temperature (RT). The slides were stained with 5% aqueous AB stain (Merck, Germany) in 4% acetic acid (pH = 3.5) for 10 min and rinsed twice with distilled water. Finally, 100 sperms were counted in different areas of each slide using a light microscope ( × 1000 magnification). Unstained or pale blue stained sperm were reported to be normal spermatozoa (AB ${}^{-}$ ), and dark blue stained sperm as abnormal spermatozoa (AB ${}^{+}$ ) (17).

#### TB staining

TB is a metachromatic dye that has a high affinity with free phosphate groups of DNA strands. Low-density abnormal chromatin was determined via this staining. First, air-dried sperm smears were placed in a fixative solution including 96% ethanol and acetone (1:1) at 4 C for 30 min. Then, the slides were incubated in 0.1 N hydrochloric acid at 4 C for 5 min and then were washed 3 times with distilled water for 2 min. Subsequently, the slides were stained with 0.05% TB (Merck, Germany) in 50% citrate phosphate (pH = 3.5) for 10 min. 100 spermatozoa were counted under a light microscope. Light blue sperm heads were considered to indicate good chromatin status, dark blue as mild abnormal chromatin, and violet and purple as severe chromatin abnormality. The spermatozoa with light blue heads were scored as normal cells (TB ${}^{-}$ ) and the rest as abnormal cells (TB ${}^{+}$ ) (18).

#### CMA3 staining 

CMA3 (Sigma, St. Louis, MO, USA) is a guanine-cytosine specific fluorochrome used for indirect measures of sperm protamine deficiency. The dried smears were fixed in Carnoy's solution at 4 C for 10 min. The slides were treated for 10 min with 100 ml of CMA3 in McIlvainʼs buffer (0.25 mg/ml) at RT with dark conditions. Then, the slides were rinsed in McIlvainʼs buffer and mounted by DPX medium. At least 100 spermatozoa were counted using fluorescent microscopy at 390-490 nm wavelength ( × 1000 magnification). This staining was used to evaluate spermatozoa where bright yellow (CMA3 ${}^{+}$ ) was considered abnormal, and yellowish-green (CMA3 ${}^{-}$ ) was considered to indicate sperm with normal protamination (18).

#### Evaluation of sperm apoptosis by TUNEL assay

TUNEL assay was used to calculate the percentage of apoptotic spermatozoa. The TUNEL kit was an in-situcell death detection kit (Roche Diagnostics, Mannheim, Germany) that detected single or double-stranded fragmented DNA tagged to label nucleotides in a reaction catalyzed by the enzyme TdT. In accordance with the kit protocol, the smears were fixed in 100% methanol for 30 min and then were rinsed in PBS at RT. The slides were incubated with blocking solution for 15-20 min at 25 C in dark conditions. After being washed with PBS, they were incubated with 0.1% Triton X-100 (Merck, Germany) and 0.1% sodium citrate for 5 min on ice. After that, staining with TUNEL solution was carried out for 1 hr in a dark and humid environment at 37 C. The slides were washed 3 times and analyzed by a fluorescence microscope. Sperms with bright green heads (TUNEL ${}^{+}$ ) had fragmented DNA, and sperms with pale green heads (TUNEL ${}^{-}$ ) had healthy DNA (19).

### Evaluation of sperm membrane by LPO assay 

The LPO kit (Cat: AB133085, Abcam, UK) measures hydroperoxides utilizing redox reactions with ferrous ions. Lipid hydroperoxides must be extracted from the sample into chloroform before performing the assay. Then, 500 μl of chloroform extract, 450 μl of chloroform-methanol solvent, and 50 μl of chromogen solution (equal volumes of FTS reagent 1 and FTS reagent 2) were mixed in the sample test tubes. Finally, the samples were incubated at RT for 5 min and read on a spectrophotometer at 500 nm (Microplate reader, Epoch, BioTek, USA).

### Mitochondrial activity evaluation with JC1 assay 

Sperm mitochondrial activity was evaluated by 5, 5', 6, 6'-tetrachloro-1-1',3,3'-tetraethyl-benzami-dazolocarbocyanin iodide known as JC-1 kit (Cayman, Item NO:10009172). In this assay, an equal volume of sperm suspension was mixed with JC-1 solution (concentration of 50 times diluted) for 30-40 min at 37 C in dark conditions. Then, the suspension of the cells was centrifuged at 400 g for 5 min and analyzed by a fluorescence microscope (Olympus Co., Tokyo, Japan) at x1000 magnification. In cells with healthy mitochondria, JC-1 formed complexes known as J-aggregates, and the midpiece was seen in red, while in cells with low MMP, JC-1 remained in a monomeric form and was seen in green.

### Ethical considerations

All couples completed informed consent forms, and also this study was approved by the Ethics Committee of Yazd Reproductive Sciences Institute, Yazd, Iran (Code: IR.SSu.MEDICINE.REC.1397.045).

### Statistical analysis

The statistical analysis was performed using the Statistical Package for the Social Sciences software version 26 (SPSS, Inc., Chicago, IL, USA). GraphPad Prism version 8.4.2 (GraphPad Software, Inc., San Diego, CA, USA) was used to draw the graphs. Normality of distribution was tested by the Shapiro-Wilk test. Data are shown as mean ± standard deviation (SD). Continuous variables that were normally distributed were assessed using one-way ANOVA with Tukey's post-hoc or Kruskal-Wallis with the Mann-Whitney U test to determine the statistical significance between the 3 groups. Significance values were adjusted by the Bonferroni correction for multiple tests. Multivariable logistic regression models were used to determine the association between the fertilization outcome and the dependent variables. P < 0.05 was considered significant.

## 3. Results

**Figure 1:**
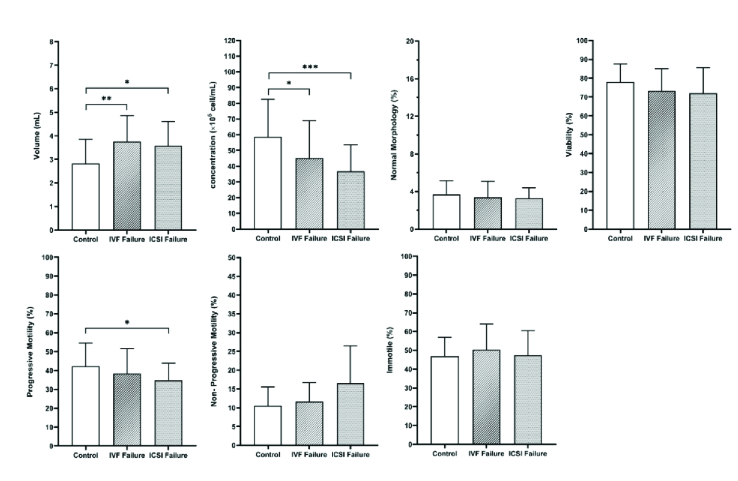
Comparison of sperm parameters in the semen of the control, IVF, and ICSI failure groups. *P < 0.05, **P < 0.01, ***P < 0.001. IVF: In vitro fertilization, ICSI: Intracytoplasmic sperm injection.

In the present study, the mean duration of marriage and of infertility were significantly different in the control vs. the experimental groups with complete failure in fertilization. Semen parameters in the 3 groups showed that concentration and semen volume (ml) were significantly different in the IVF and ICSI fertilization failure groups. Progressive motility (%) was significantly different in the ICSI failure fertilization group compared to the control group, and other parameters were not significantly different between the 3 groups (Table I and Figure 1).

**Figure 2:**
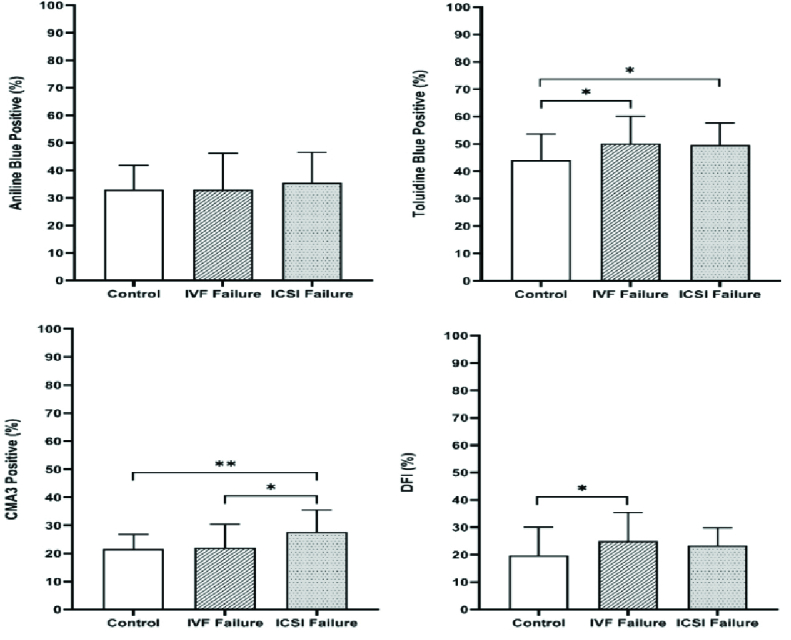
Comparison of sperm chromatin and DNA integrity status in the semen of the control, IVF, and ICSI failure groups. *P < 0.05, **P < 0.01. IVF: In vitro fertilization, ICSI: Intracytoplasmic sperm injection, DF: DNA fragmentation.

**Figure 3:**
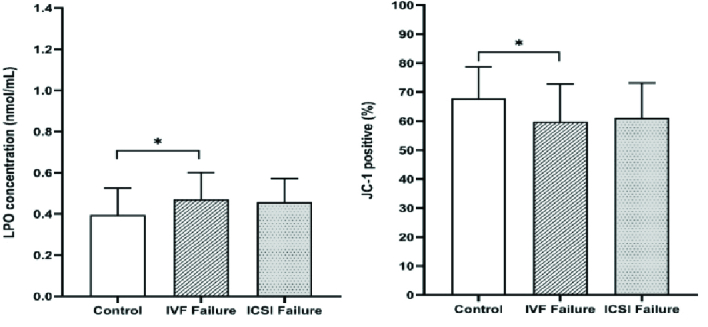
Comparison of LPO concentration and of JC1 positive (%) in the semen of the control, IVF, and ICSI failure groups. *P < 0.05. IVF: In vitro fertilization, ICSI: Intracytoplasmic sperm injection, LPO: Lipid hydroperoxides.

Regarding sperm chromatin and DNA integrity status, the data showed that there were statistically significant differences between the 3 groups obtained from the TB (p = 0.01), TUNEL (p = 0.02), and CMA3 (p < 0.001) tests, but not from the AB staining (Table II and Figure 2). Also, LPO concentration and high MMP (JC1 positive) showed statistically significant differences between the IVF fertilization failure group compared to the control group (p = 0.04 and p = 0.02, respectively) (Figure 3). For further evaluation about the effects of the variables on fertilization outcome, logistic regression was done. The association of sperm parameters and chromatin/DNA status with TFF were surveyed in the IVF failure and ICSI failure groups. However, the logistic regression model showed that the above-mentioned parameters, except for sperm viability in the ICSI failure group (odds ratio = 0.876, 95% confidence interval = 0.781-0.982), were not predictors for the fertilization outcome in the experimental groups.

**Table 1:** The results of the sperm parameters in the 3 groups


**Variables**	**IVF fertilization failure**	**ICSI fertilization failure**	**Control**	**P-value**
**Semen volume (ml)**	3.76 ± 1.09	3.57 ± 1.04	2.83 ± 1.02	< 0.001*
**Concentration (x 10^6^/ml)**	45.10 ± 23.83	36.67 ± 16.97	58.83 ± 23.58	< 0.001*
**Morphology (%)**	3.40 ± 1.67	3.40 ± 1.67	3.30 ± 1.08	0.4*
**Viability (%)**	73.37 ± 11.68	71.97 ± 13.52	78.03 ± 9.45	0.11**
**Progressive motility (%)**	38.43 ± 13.11	34.90 ± 8.88	42.33 ± 12.17	0.04**
**Non-progressive motility (%)**	11.60 ± 5.04	16.60 ± 9.86	10.56 ± 5.01	0.06*
**Immotile (%)**	50.30 ± 13.74	47.43 ± 13.11	46.97 ± 10.05	0.39*
Data presented as Mean ± SD. *Kruskal-Wallis test, **One-way ANOVA, IVF: In vitro fertilization, ICSI: Intracytoplasmic sperm injection				

**Table 2:** The results of sperm chromatin and DNA status in the 3 groups


**Variables**	**IVF fertilization failure**	**ICSI fertilization failure**	**Control**	**P-value**
**Aniline blue (%)** ${}^{*}$	32.97 ± 13.18	35.47 ± 11.03	33 ± 8.89	0.61
**Toluidine blue (%)** ${}^{*}$	50.23 ± 9.93	49.70 ± 7.94	44 ± 9.69	0.01
**Chromomycin A3 (%)** ${}^{*}$	22.03 ± 8.34	27.67 ± 7.84	21.5 ± 5.29	< 0.001
**TUNEL (%)** ${}^{**}$	25.03 ± 10.42	23.33 ± 6.54	19.67 ± 10.37	0.02
Data presented as Mean ± SD. *One-way ANOVA, **Kruskal-Wallis test. IVF: In vitro fertilization, ICSI: Intracytoplasmic sperm injection; TUNEL: Terminal deoxynucleotidyl transferase-mediated dUTP nick end labeling				

## 4. Discussion

This study showed a significant differences in sperm parameters, including progressive motility, between the ICSI fertilization failure group and the control group. Also, all chromatin/DNA integrity tests found significant differences between the 3 groups except for AB staining. Interestingly, the ROS level was higher and MMP was lower in the IVF group compared to the control group. However, logistic regression demonstrated that none of the DNA status assays were a predictor for the fertilization outcome in the experimental groups.

TFF in IVF and ICSI can happen for a variety of reasons. In this study, cases with TFF had experienced a failure in fertilization in either IVF or ICSI cycles. In the present study, the mean duration of marriage and of infertility were significantly different in the control group vs. the cases. Previous studies have shown that longer infertility duration can be correlated with the incidence of TFF (1). Unexplained fertilization failure can be associated with oocyte activation failure, immunology defects, genetic and meiotic errors or hardening of the zona pellucida, which can increase over time (20). Participants with longer infertility durations were a high-risk population for TFF.

Sperm parameters may be used in the first stage of diagnosis to predict fertilization results. The mean sperm volume and concentration were significantly lower in both the IVF and ICSI failure groups compared to the control group, although the sperm count remained within a normal range. Previous studies have concluded that the fertilization rate can increase with high progressive motile sperm and sperm concentration, while others have found that morphology was not associated with TFF (10).

Our findings showed that sperm progressive motility was significantly different between the ICSI group and the control group, while non-progressive motility, viability and morphology of sperm were no different. Previous studies have reported a low number of progressive spermatozoa as a predictive factor for TFF (21). On the other hand, progressive motility was not significantly different between the IVF group and the control group. In our study, the logistic regression model showed that sperm viability was predictive for fertilization failure in the ICSI group. Also, sperm parameters were not sufficient to predict fertilization failure in the IVF cycle. In line with our study, a previous study found that a lower number of oocytes retrieved and a reduction in the interaction of sperm and zona pellucida were the main causes of induced fertilization failure in an IVF program (22). According to our study, sperm morphology was not different in the IVF or ICSI groups. Contrary to this finding, it has been reported that cases with a low percentage of morphologically normal sperm ( < 4%), and low sperm concentration and motility are at a high risk of fertilization failure (23). The findings on the effect of sperm morphology in IVF and ICSI cycles have been inconsistent. Using more advanced methods and high magnification (x6600), including using motile sperm organelle morphology examination, can help to better evaluate sperm morphology (24). Despite the widespread use of visual examination, it is limited in determining male infertility and assisted reproductive outcomes. Molecular testing, as a more robust tool for the assessment of sperm quality, is needed for diagnosis. AB and TB assays are useful and simple tests for routine diagnosis of male infertility.

In the present study, the results of chromatin abnormalities found that the mean percentage of sperm with extra histones in the control group and both groups of participants with complete failure in fertilization were not significantly different. Although the number of excess histones in the 3 groups was not significantly different, we saw an average increase in the number of excessive histones in the IVF and ICSI fertilization failure groups. CMA3, TUNEL, and TB staining were applied to evaluate sperm chromatin structure, and the level of apoptosis showed significant differences in the 3 groups. Previously, researchers have evaluated the effects of sperm chromatin integrity on ICSI outcomes. They reported no significant correlation between the results of chromatin assays, including acridine orange, AB, TB and CMA3, and fertilization outcomes, like our study (25). Also, others have found no significant correlation between chromatin integrity defects and fertilization rate in infertile couples undergoing IVF/ICSI (26). Neither sperm fragmentation nor high DNA stainability scores detected by acridine orange stain can provide independent information about embryo quality and fertilization rates in ICSI cycles.

On the other hand, contrary to our study, others showed a significant negative correlation between protamine deficiency and fertilization rate in ICSI cycles (11). They reported that CMA3 staining was a sensitive and useful tool to assess fertilization potential before the ICSI procedure. Therefore, there are still contradictory opinions about the effect of sperm chromatin status on fertilization rate, embryo quality, and pregnancy outcomes. It has been shown that sperm DNA damage can be correlated with IVF failure. This may be due to oocytes that prevent the entry of sperm with a high degree of DNA damage. Fertilization naturally occurs in the ICSI method with high sperm DNA damage because at the time of fertilization and a few days after that, there is no need to express paternal genes, and sperm DNA damage during the first days seems normal (5). According to our study, although sperm parameters were not different in the IVF process, DNA evaluation showed a significant difference between the IVF fertilization failure group and the control group. Evaluation of DNA fragmentation by TUNEL assay may help predict fertilization rate in IVF programs.

ROS is an important factor that in low levels causes normal fertilization; however, in higher levels, it leads to destructive effects including membrane LPO, protein modification, DNA damage, zona pelucida hardening, and ATP depletion. High levels of ROS in semen have been associated with poor morphology, motility, and low sperm count. Furthermore, LPO is damaged by ROS and may impair sperm function, damage MMP, decrease the acrosome reaction and lead to TFF (27, 28). The amount of LPO in the IVF group with complete failure in fertilization was significantly higher than in the control group (p = 0.04). MMP was significantly lower in the IVF fertilization failure group compared to the control group.

Previous studies have shown that both high MMP and low DNA fragmentation of spermatozoa are correlated with forward motility and higher fertilization rates after IVF (29, 30). In conventional IVF, the oocytes, cumulus cell mass, and spermatozoa used for insemination (150-200 × 10^3^) are a potential source to increase ROS levels, while there is no accumulation of sperm in ICSI.

## 5. Conclusion

The data showed that high ROS and low MMP levels may have detrimental effects on sperm fertility in IVF. The sperm with normal parameters may still have high DNA fragmentation damage, causing TFF in IVF and ICSI programs. In addition, the logistic regression analysis revealed that sperm viability was predictive for fertilization failure in the ICSI group. Sperm chromatin and DNA quality assays were not predictors for TFF in either group. Performing diagnostic tests, especially in cases with previous fertilization failure, can reduce the risk of TFF.

##  Conflict of Interest

The authors declare that there is no conflict of interest.
